# Persistence of self-injurious behaviour in autism spectrum disorder over 3 years: a prospective cohort study of risk markers

**DOI:** 10.1186/s11689-016-9153-x

**Published:** 2016-05-05

**Authors:** Caroline Richards, Jo Moss, Lisa Nelson, Chris Oliver

**Affiliations:** Cerebra Centre for Neurodevelopmental Disorders, School of Psychology, University of Birmingham, Edgbaston, B15 2TT UK; Institute of Psychiatry, King’s College London, London, UK

**Keywords:** Autism spectrum disorder, Self-injury, Risk marker, Prevalence, Hyperactivity, Impulsivity, Pain

## Abstract

**Background:**

There are few studies documenting the persistence of self-injury in individuals with autism spectrum disorder (ASD) and consequently limited data on behavioural and demographic characteristics associated with persistence. In this longitudinal study, we investigated self-injury in a cohort of individuals with ASD over 3 years to identify behavioural and demographic characteristics associated with persistence.

**Methods:**

Carers of 67 individuals with ASD (Median age of individuals with ASD in years = 13.5, Interquartile Range = 10.00–17.00), completed questionnaires relating to the presence and topography of self-injury at T_1_ and three years later at T_2_. Analyses were conducted to evaluate the persistence of self-injury and to evaluate the behavioural and demographic characteristics associated with persistence of self-injury.

**Results:**

At T_2_ self-injurious behaviour had persisted in 77.8 % of individuals. Behavioural correlates of being non-verbal, having lower ability and higher levels of overactivity, impulsivity and repetitive behaviour, were associated with self-injury at both time points. Risk markers of impulsivity (*p* = 0.021) and deficits in social interaction (*p* = 0.026) at T_1_ were associated with the persistence of self-injury over 3 years.

**Conclusions:**

Impulsivity and deficits in social interaction are associated with persistent self-injury in ASD and thus may act as behavioural risk markers. The identification of these risk markers evidences a role for behaviour dysregulation in the development and maintenance of self-injury. The findings have clinical implications for proactive intervention; these behavioural characteristics may be utilised to identify ‘at risk’ individuals for whom self-injury is likely to be persistent and therefore those individuals for whom early intervention may be most warranted.

## Background

Self-injurious behaviour is common in autism spectrum disorder (ASD), with estimates ranging from 35 to 60 % [1–5]. These prevalence estimates are significantly higher than those reported for populations with intellectual disability of heterogeneous aetiology [6–8]. The presence of self-injury leads to a higher risk of psychiatric hospitalisation [9], reactive physical intervention [10] and lower quality of life [11]. Carers and staff of those who display self-injury are reported to experience higher levels of stress and burnout [12–14]. In addition to the personal costs of self-injury, there are also significant financial costs to services [15]. Behavioural interventions for self-injury, which employ function-based differential reinforcement procedures, are effective but are often resource intensive [16–18]. Consequently, attention has turned to the viability of a targeted early intervention strategy for self-injurious behaviour [19, 20], which is predicated on the assumption that self-injury begins during childhood/early adulthood, becomes more severe with time and persists over time [21–23].

Estimates of the persistence of self-injury in individuals with intellectual disabilities vary considerably. However, the majority of studies demonstrate that self-injury can be persistent in individuals with intellectual disability with large longitudinal studies reporting 71 % persistence over 7 years (age range at follow-up = 12–65 years) [23] and 84 % persistence over 20 years (age range at follow-up = 24–82 years) [22]. However, there are also a number of studies which appear to show some remission in self-injury. Murphy and colleagues demonstrated that the prevalence of self-injury significantly decreased over time (age range at follow-up = 13.5–30.4 years) [24]. Similarly, Cooper and colleagues reported a moderate two year remission rate in adults with intellectual disability (38.2 %; mean and *SD* age in years at follow-up = 43.6; *14.2*) [25]. However, despite presenting evidence that self-injurious behaviour may not be as persistent as initially thought, the data reported by Cooper and colleagues reveal that 61.8 % of adults continued to show self-injurious behaviour that caused tissue damage, was pervasive, presented significant risks to the health or safety of the person and significantly impacted upon their own or other’s quality of life. Thus, these data still suggest that for the majority of individuals, self-injury continues to be a behaviour which significantly and persistently impacts upon their lives.

Despite the high prevalence of self-injury in ASD, few studies have examined persistence. A recent literature review suggests that challenging behaviours may be stable in individuals with ASD [26]. Cross-sectional designs offer convergent evidence that prevalence of self-injury remains constant across subsamples of young children, children and young adolescents with ASD [27]. However, in studies with longitudinal designs of adolescents and adults with ASD [5] and children with pervasive developmental disorders (PDD) [28], the presence of self-injury decreased significantly over 4.5 and 3 years, respectively. However, both studies recruited through service agencies, clinics and hospitals where individuals were receiving treatment. Therefore, it is not possible to evaluate whether the reported remission in self-injury in ASD is the natural course of development or a result of intervention. Additionally, the sample recruited by Baghdadli and colleagues were young (mean age at follow-up 8 years), and thus, self-injury behaviour may not yet have become established. A 7-year follow-up of the cohort described by Baghdadli and colleagues [2], reported that 35.8 % of the sample showed self-injury, a similar prevalence figure to that reported originally by Baghdadli and colleagues (32.7 %). Whilst persistence was not evaluated statistically in the 7-year follow-up, the comparable prevalence figures in the two studies suggest that self-injury may have persisted. Thus, there remains a need to evaluate statistically the persistence of self-injury in a population with ASD that have not been recruited from clinical services. If persistence is high, then in combination with models of the development and maintenance of self-injury this would allude to the importance of early intervention.

Proactive intervention strategies to reduce negative outcomes have been implemented effectively in physical health settings [29] and more broadly in early intensive behavioural interventions for autism [30–32]. It is hoped that by providing interventions for socially maintained self-injury when individuals with ASD are young, outcomes will be more positive as reinforcement history for self-injury will be shorter and, consequently, the behaviours will be less resistant to change [21]. In order for early intervention strategies to be effective, it would be beneficial to identify those individuals with the greatest risk of developing self-injury that is likely to be persistent as opposed to transient. Delineating behavioural correlates (characteristics associated with the presence of self-injury *at a single time point*) and risk markers (characteristics associated with the persistence of self-injury *over time*) could aid early intervention thorough strategic targeting of interventions towards those who evidence the risk markers for persistent self-injury at an earlier stage in the development of the behaviour.

There is emerging evidence of demographic and behavioural characteristics that are associated with self-injury in ASD at a single time point. These putative risk markers include impairments in adaptive skills [1–3], greater severity of autism [1, 2, 33], younger age [34], perinatal conditions [1] and repetitive and impulsive behaviours [3, 4]. Studies of populations of individuals with intellectual disabilities have identified lower ability [25], lower verbal ability [35], attention deficit hyperactivity disorder [25], visual impairment [25] and the site of self-injury [23, 35] as variables which independently predict the persistence of self-injury over time. Two studies have evaluated the predictive value of variables to identify persistent self-injury in ASD [5, 28]. Speech deficits, autism severity, intellectual disability and older age were all identified as risk markers. However, these studies were not able to link these risk markers to persistent self-injury as self-injury was either grouped into an outcome subscale with repetitive, withdrawn and inattentive behaviours [5] or both onset of self-injury and persistence of self-injury were grouped into a ‘negative outcome’ category [28]. Thus, there remains a need to identify risk markers associated with persistent self-injury in individuals with ASD.

In summary, the prevalence of self-injury has been reliably demonstrated to be elevated in those with ASD compared to those with intellectual disability of heterogeneous aetiology [1, 3, 5, 36]. There is an evidence that self-injury is persistent in the majority of individuals with intellectual disability; however, the data in individuals with ASD are equivocal [5, 23]. Prior to a consideration of early intervention and putative risk markers for self-injury in ASD, evidence should be gathered regarding the persistence of self-injury. In order to guard against threats to external validity, these data should be drawn from a population with ASD that has not been recruited from a clinical sample. There is evidence associating a range of demographic and behavioural characteristics with self-injury in ASD at a single time point [1, 34] and predicting persistent self-injury in intellectual disability populations [23, 25, 35]. However, there is currently very limited study in ASD of characteristics associated with self-injury at multiple time points and associated with persistent self-injury [5, 28]. These data could contribute to a targeted early intervention strategy. In this study, we conduct longitudinal assessments of a sample of individuals with ASD. A sub-group of this sample at the first time point of this study were reported by [3]. At the first time point, self-injury was associated with significantly higher levels of impulsivity, hyperactivity and negative affect and significantly lower levels of adaptive ability and speech. The present follow-up study has these aims:

i) To compare prevalence, topographies and severity of self-injury at (T_1_) and (T_2_) 3 years later, to establish the persistence of self-injury.
ii) To investigate behavioural and demographic variables associated with self-injury at T_2_. We predicted that certain demographic and behavioural variables associated with self-injury in the longitudinal sample at T_1_ and/or in previous literature (poor speech, impulsivity, overactivity, repetitive behaviours, autism spectrum disorder phenomenology) will also be associated with self-injury at T_2_.
iii) To evaluate the value of these behavioural and demographic variables at T_1_ to differentiate between absent, transient and persistent self-injury at T_2_.

## Methods

### Recruitment

Participants with ASD were recruited in the UK via the National Autistic Society at T_1_. These participants were contacted and invited to participate 3 years later at T_2_. In total, 190 participants were invited to take part at T_2,_ and 68 carers of individuals with ASD completed the assessments (return rate 35.78 %). The average follow-up time was 36.4 months (range = 34–39 months).

### Procedure

Carers received an information sheet, cover letter, consent form, demographic questionnaire and questionnaire pack. To avoid priming, the study was described as investigating behaviours associated with ASD. Carers returned completed questionnaires and consent forms in a prepaid envelope.

Ethical approval for this study was obtained from the ethical review committee at the University of Birmingham

### Participants

Participants were excluded if at T_1_, (1) they were under the age of four as some measures were not appropriate, (2) they did not have a confirmed diagnosis of ASD from a relevant professional (Psychiatrists, Clinical Psychologists, Educational Psychologists, General Practitioner, Clinical Geneticist or Paediatrician) as reported by parents/carers, (3) they had an additional diagnosis of a comorbid genetic syndrome, (4) they had incomplete total scores on the Social Communication Questionnaire (SCQ) [37], and (5) they scored below the ASD cutoff on the SCQ. If a large proportion of the data at T_1_ or T_2_ were incomplete (25 % or more of items across questionnaires), participants were also excluded. Thus, one participant was excluded from the sample.

A sub-group of the T_1_ sample was reported on by Richards and colleagues [3]. This sub-group comprised only those who had an associated intellectual disability, defined by proxy via a score of less than nine on the self-help subscale of the Wessex [38]. However, in order to ensure a large and representative sample at follow-up, the whole sample (those with a score of less than nine *and* those with a score of nine on the self-help subscale) was invited to take part at T_2_.

To ensure that the T_2_ sample was representative of the T_1_ sample, and not biased by 122 participants who declined to take part T_2_, a series of Mann-Whitney *U* and *χ*^2^ analyses were conducted to detect possible significant differences between participants included at T_2_ (67) and those from the T_1_ sample who were not included. Table 1 describes the demographic and behavioural characteristics of those who took part at T_2_ and those who declined to take part at T_2_

**Table 1:** T_1_ Demographic characteristics comparing participants at T_2_ with those who declined to participate at T_2_

		Took part at T_2_	Declined to take part at T_2_	Mann-Whitney *U*/*χ* ^2^	*df*	*p* value^a^
*N*		67	122			
Age	Median (IQR)	10.00 (7.00–14.00)	10.00 (7.00–14.00)	4287.50	–	0.576
Gender	% male	85.1	86.1	0.35	1	0.852
Self help	% partly able/able^b^	89.6	85.2	0.70	1	0.403
Mobility	% mobile	98.5	95.1	N/A^c^	–	0.425
Speech	% verbal	89.6	82.8	1.30	1	0.255
Self-injury	% with behaviour	*40.3*	*54.9*	*3.92*	*1*	*0.048*
Mood^d^ total score	Median (IQR)	34.00 (30.00–38.00)	32.00 (27.00–38.00)	4470.00	–	0.287
Activity^e^ total score	Median (IQR)	44.00 (24.63–53.00)	44.00 (30.00–56.00)	3566.00	–	0.172
Repetitive behaviour^f^ total score	Median (IQR)	28.00 (17.00–37.00)	29.50 (17.10–40.00)	3748.00	–	0.443
ASD phenomenology^g^ total score	Median (IQR)	28.00 (23.00–31.00)	26.00 (22.00–30.12)	3722.5	–	0.310

^a^Significant differences are highlighted in italics (*p* < 0.05; two tailed)

^b^Based on the self-help scale of the Wessex Behaviour Schedule. Able or partly able is defined as a score of >2

^c^Fishers exact was calculated as two cells had an expected count of <5

^d^Mood, Interest and Pleasure Questionnaire

^e^The Activity Questionnaire

^f^Repetitive Behaviour Questionnaire

^g^Social Communication Questionnaire

The analysis revealed that significantly more individuals without self-injury took part at T_2_ than individuals with self-injury. Apart from this, individuals who took part at T_2_ did not differ on any other demographic or behavioural variable, to the individuals who declined to take part at T_2_. This suggests that the data sample collected at T_2_ is broadly representative of the original sample collected at T_1_.

### Measures

The questionnaire pack included the following informant based questionnaire measures which are appropriate for children and adults with intellectual disabilities.

A demographic questionnaire to collect information on gender, mobility, verbal ability and diagnosis.

The Wessex [38] was used to assess ability in children and adults with intellectual disabilities. It comprises five subscales including continence, mobility, self-help skills, speech and literacy. For this study, the self-help subscale was used to estimate the degree of ability, and responses to items on mobility, speech, reading, writing and counting were used to further describe the groups. The Wessex Scale has no published validity data but evidences modest inter-rater reliability at subscale level for both children and adults (mean kappa value of 0.62 and 0.54 for overall classification and item level reliability, respectively) [38, 39]. The Wessex has been argued to be an effective tool for large-scale questionnaire studies [39].

The Mood Interest and Pleasure Questionnaire-Short form (MIPQ-S) [40] assesses affect and comprises 12 items, forming two subscales: mood and interest and pleasure. The measure has good internal consistency (Cronbach’s alpha coefficients: total = 0.88, mood = 0.79, interest and pleasure = 0.87), test-retest (0.97) and inter-rater reliability (0.85). Internal consistency for subscales is good (alpha coefficient range for subscales 0.84–0.94). Concurrent validity between the MIPQ and the Aberrant Behavior Checklists’s (ABC) ranged from medium to strong (0.36–0.73; *p* < 0.001).

The Activity Questionnaire (TAQ) [41] was included to assess behaviours indicative of overactivity and impulsivity. The measure has 18 items which form three subscales of overactivity, impulsivity and impulsive speech. The TAQ has no published validity data but evidences item-level inter-rater reliability from 0.31 to 0.75 (mean 0.56) and test-retest reliability ranges from 0.60 to 0.90 (mean 0.75). Inter-rater and test-retest reliability indices for subscales and total score exceed 0.70. Internal consistency for the subscales is good (alpha coefficient range for subscales 0.67–0.94).

The Social Communication Questionnaire (SCQ) [37] was included to assess ASD behaviours. The SCQ was developed as a tool for screening for ASD in children and adults and is based on the Autism Diagnostic Interview [42]. The measure consists of 40 items grouped into three subscales: communication; social interaction and repetitive and stereotyped patterns of behaviours. The authors identify a cutoff score of 15 as indicative of ASD and a higher cutoff of 22 to differentiate between individuals with autism and those with other pervasive developmental disorders. The SCQ shows good concurrent validity with the Autism Diagnostic Interview and the Autism Diagnostic Observation Schedule [43]. Internal consistency is also good (*α* = 0.90 for the total scale). Whilst at T_1_, the Lifetime Version of the Social Communication Questionnaire (SCQ) was employed [37], at T_2_, the Current Version was administered as this version is recommended in order to evaluate measurement of change over time. All analyses conducted utilising the SCQ excluded item 17 (‘has she/he ever injured her/himself deliberately, such as biting her/his arm or banging her/his head?’) to prevent confounds in self-injury analysis.

The Repetitive behaviour Questionnaire (RBQ) [44] comprises five subscales: stereotyped behaviour, compulsive behaviour, insistence on sameness, restricted preferences and repetitive speech. Previous examination of the psychometric properties of the RBQ [44] reveals good inter-rater reliability coefficients (range 0.46–0.80), test-retest reliability (range 0.61–0.93) and internal consistency (alpha coefficient range for subscales 0.50–0.78). Concurrent validity and content validity between the RBQ and the repetitive behaviour subscale of the ASQ are good (0.6; *p* < 0.001).

The Challenging Behaviour Questionnaire (CBQ) [45] was derived directly from the Challenging Behaviour Interview [46]. The CBQ evaluates the presence of self-injury, physical aggression, verbal aggression, destruction of property and stereotyped behaviour in the last month. The measure also examines eight topographies of self-injurious behaviour that were adapted from Bodfish and colleagues [47]. These items are as follows: hits self with body part, hits self against surface or object, hits self with object, bites self, pulls, rubs or scratches self, inserts finger or objects. The CBQ also asks about any other forms of self-injury the individual may show and asks the caregiver to specify what these topographies are. Items evaluating self-injury were used for the current study. Previous examination of the psychometric properties of the questionnaire has demonstrated good inter-rater reliability with reliability coefficients ranging from 0.61 to 0.89 [45]. Concurrent validity between the CBI and the Aberrant Behavior Checklists (ABC) ranged from medium to strong (0.19–0.68; *p* < 0.050 [46].

The order of the measures in the questionnaire pack was counterbalanced.

### Data analysis

Data were tested for normality using Kolmogorov-Smirnov tests. Where data were not normally distributed (*p* < 0.05), logarithmic and square root transformations were applied in order to normalise the data. However, these were not successful in changing the distribution and therefore non-parametric analyses were employed. McNemar tests were conducted in order to examine the persistence and topographies of self-injury. A self-injury severity score was calculated by summing items regarding the length of time self-injury was displayed for, the frequency of self-injury, and the level of intervention required for self-injury. The Wilcoxon signed ranks test was used to evaluate differences in this score between T_1_ and T_2_.

Chi-square, relative risk statistics and Mann-Whitney *U* tests were conducted in order to examine the difference between those who engaged in self-injury and those who did not on a variety of demographic and behavioural characteristics. Kruskal Wallis tests were employed to test for differences in T_1_ putative risk markers between absent (self-injury absent at both T_1_ and T_2_), transient (self-injury present at *either* T_1_ or T_2_) and persistent (self-injury present at both T_1_ and T_2_) self-injury groups. Chi-square and Fisher’s exact *t* tests were used to test for these differences in categorical data.

## Results

### Changes in demographic and behavioural characteristics over time

Prior to analysis, the demographic and behavioural characteristics of the sample included at T_2_ were compared to the demographic and behavioural characteristics of the same sample at T_1_. This was done in order to evaluate whether any changes had occurred in demographic and behavioural characteristics that may interact with the persistence of self-injury. Table 2 presents the demographic and behavioural characteristics of the sample included at T_2_ and T_1_. In order to test for differences between the two time points, Wilcoxon signed ranks tests were conducted for the continuous data, and McNemar analyses were conducted for the categorical data.

**Table 2:** Demographic and behavioural characteristics of the selected sample at T_1_ and T_2_

		T_1_	T_2_	*p* value^a^
*N*		67	67	
Age	Median (IQR)	10.00 (7.00–14.00)	13.50 (10.00–17.00)	*<0.001*
Self help	% partly able/able	89.6	88.1	1.00
Mobility	% mobile	95.5	97.0	1.00
Speech	% verbal	95.5	91.0	0.50
Vision	% normal	97.0	86.6	0.39
Hearing	% normal	98.5	98.5	1.00
MIPQ total score	Median (IQR)	34.00 (30.00–38.00)	34.00 (29.00–40.00)	0.264
TAQ total score	Median (IQR)	44.00 (24.63–53.00)	41.00 (21.00–50.00)	0.083
RBQ total score	Median (IQR)	28.00 (17.00–37.00)	26.00 (18.00–33.00)	0.289
SCQ total score	Median (IQR)	28.00 (15.00–37.00)	21.36 (6.00–34.00)	*<0.001*
SCQ total self-injury	Median (IQR)	30.00 (18.00–37.00)	25.00 (11.00–34.00)	*<0.001*
SCQ total no self-injury	Median (IQR)	26.00 (15.00–35.00)	17.00 (6.00–30.00)	*<0.001*

^a^Significant differences between the two data collection points are highlighted in italics (*p* < 0.01; all tests are two tailed apart from age)

The results presented in Table 2 reveal no significant differences for demographic or behavioural characteristics between T_1_ and T_2_. There was a significant difference between SCQ scores at T_1_ and T_2_. This difference was significant for both the self-injury and non-self-injury group at T_2_.

### Persistence of self-injury, topographies of self-injury and severity of self-injury

In order to examine the persistence, remission and incidence of self-injury, the percentage of the sample who showed self-injurious behaviour, and the various topographies of self-injury, was calculated (see Table 3). McNemar analysis was employed to assess the persistence of self-injury.

**Table 3:**
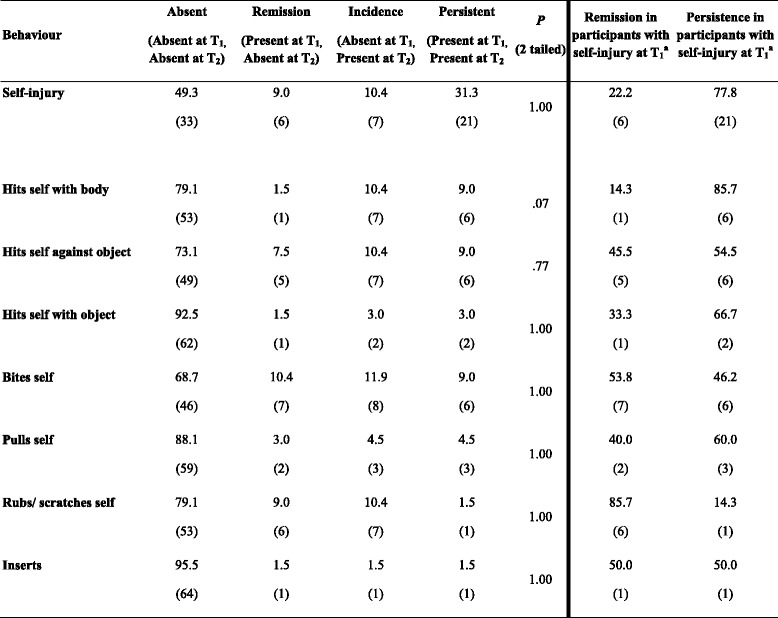
Percentage (point prevalence) of participants showing remission, incidence, persistence and no self-injurious behaviour

^a^Remission and persistence of self-injurious behaviour in participants showing the behaviour at T_1_ (right of the bold line)

The results presented in Table 3 reveal no significant differences in the presence or topography of self-injury displayed at T_1_ and T_2_, indicating that the behaviour is persistent and stable over time. In order to evaluate the stability of the severity of self-injury, the self-injury severity score at T_1_ (median = 6.00, interquartile range = 4.00–8.00) and the self-injury severity score at T_2_ (median = 5.00, interquartile range = 4.00–7.50) of those with persistent self-injury were compared using Wilcoxon signed ranks test. The results revealed no significant difference between the self-injury severity scores at T_1_ and T_2_ (*N* = 21, *p* = 0.374). Cumulative incidence was also calculated and was found to be 7 cases per 40 individuals or 17.5 % over 36.4 months. In summary, the results revealed that the presence, topography and severity of self-injury were persistent and stable over three years.

### Demographic and behavioural characteristics associated with self-injury at T_2_

In order to examine the behavioural correlates associated with self-injury, comparisons were made between those who displayed self-injury and those who did not at T_2_ on a variety of demographic and behavioural characteristics. Table 4 reports the differences between those with self-injury and those without on demographic measures.

**Table 4:** Demographic variables for participants with self-injury and without self-injury at T_2_

		Percentage of individuals with self-injury (*N*—point prevalence)	Percentage of individuals without self-injury (*N*—point prevalence)	Chi-square	*P* value^a^	Relative Risk (95 % CI)
Gender	Male	82.1 (23)	89.7 (35)	N/A^b^	0.474	–
	Female	17.9 (5)	10.3 (4)			
Age^c^	<11 years	39.3 (11)	38.5 (15)`	0.590	0.745	–
	12–18 years	46.4 (13)	38.5 (15)			
	≥19 years	14.3 (4)	20.5 (8)			
Ability^c^	Able/partly able	75.0 (21)	97.4 (38)	N/A^b^	***0.008***	***2.4 (1.5–3.8)***
	Not able	21.4 (6)	2.6 (1)			
Speech^c^	Verbal/partly verbal	78.6 (22)	100.0 (39)	N/A^b^	***0.005***	***2.8 (2.0–3.9)***
	Non-verbal	17.9 (5)	0.0 (0)			
Mobility^c^	Mobile	92.6 (26)	100.0 (39)	N/A^b^	0.409	–
	Non-mobile/partly mobile	3.6 (1)	0.0 (0)			
Vision	Normal	89.3 (25)	84.6 (33)	N/A^b^	0.724	–
	Poor vision/blind	10.7 (3)	15.4 (6)			
Hearing	Normal	96.4 (27)	100.0 (39)	N/A^b^	0.418	–
	Poor hearing/deaf	3.6 (1)	0.0 (0)			

^a^Significant differences (*p* < 0.05) are highlighted in italics; variables for which significant differences at *p* < 0.05 were obtained at T_1_ are set in bold (all tests are two-tailed apart from level of ability and speech)

^b^Fisher’s exact was calculated

^c^One case of missing data

The results reveal that individuals with self-injury were significantly more likely to be non-verbal than those who did not engage in self-injury. Additionally, individuals with self-injury were significantly more likely to be classified as ‘not able’ by the Wessex, as evidenced by poorer self-help skills. There were no significant differences between those who engaged in self-injury and those who did not, on any other demographic items.

Table 5 reports the differences between those with self-injury and those without, on measures of behavioural characteristics. Cliff’s dominance (or *d*) statistic [48] was used to calculate effect sizes for Mann-Whitney *U* tests.

**Table 5:** Scores and statistics for affect, repetitive behaviour, activity level and autism phenomenology for participants with and without self-injury at T_2_

Measure subscale	Median scores (interquartile range)		Mann-Whitney *U* score	*P* value^a^	Cliff’s *d*
	With self-injury (*N* = 28)	Without self-injury (*N* = 39)			
*MIPQ-S*					
Mood	18.91 (15.25–21.00)	21.00 (18.00–23.00)	***401.00***	***0.032***	0.27
Interest and pleasure	13.50 (10.00–16.75)	17.00 (12.00–19.00)	**420.00**	**0.054**	–
*RBQ*					
Stereotyped behaviour	8.00 (6.00–11.75)	5.00 (2.00–9.00)	*720.50*	*0.013*	0.32
Compulsive behaviour	8.50 (4.50–12.75)	4.00 (1.00–8.00)	*748.50*	*0.005*	0.37
Insistence on sameness	4.00 (2.25–8.00)	3.00 (0.00–6.00)	*680.00*	*0.043*	0.25
Restricted preferences^b^	6.00 (2.00–8.00)	5.00 (2.00–7.00)	426.50	0.331	–
Repetitive language^b^	7.00 (3.50–9.00)	4.00 (0.00–8.00)	496.50	0.060	–
*TAQ*					
Overactivity	22.43 (13.00–30.75)	14.00 (6.00–21.00)	***759.00***	***0.004***	0.39
Impulsivity	20.50 (16.00–22.00)	16.00 (9.00–19.00)	***745.00***	***0.006***	0.36
Impulsive speech^b^	3.00 (1.00–8.50)	5.00 (2.00–6.00)	**383.50**	**0.403**	–
*SCQ*					
Communication	8.00 (6.00–9.75)	7.00 (6.00–9.00)	631.00	0.097	–
Social Interaction	7.00 (5.00–7.00)	5.00 (3.00–6.00)	*814.50*	*<0.001*	0.49
Repetitive behaviour	9.50 (7.00–12.00)	5.00 (3.00–9.00)	*825.00*	*<0.001*	0.51

^a^Text in italics indicates a significant difference (*p* < 0.05, one tailed); variables for which significant differences at *p* < 0.05 were obtained at T_1_ are in bold

^b^Subscales only calculated for verbal participants

The results in Table 5 reveal that at T_2_, individuals with self-injury evidenced significantly lower mood and the difference in interest and pleasure approached significance. At T_2_, individuals with self-injury also evidenced significantly higher scores for measures of stereotyped behaviour, compulsive behaviour, insistence on sameness, overactivity and impulsivity. Additionally, individuals with self-injury evidenced significantly higher scores for measures of ASD phenomenology, specifically impairments in social interaction and repetitive behaviour. Small to medium effect sizes were identified for all of these differences. There were no significant differences identified for any other behavioural characteristics.

In summary, individuals with self-injury were significantly more likely to be less able and non-verbal and to show higher levels of stereotyped behaviour, compulsive behaviour, insistence on sameness, overactivity, impulsivity, repetitive behaviour and impairments in social interaction.

### Comparison of persistent, transient and absent self-injury groups on T_1_ behavioural and demographic variables

In order to evaluate the utility of the putative risk markers to differentiate between those with and without self-injury, participants at T_2_ were categorised into three self-injury groups: absent (self-injury absent at both T_1_ and T_2_; *N* = 33, mean (*SD*) age in years = 12.00 (*7.03*), % male = 90.9), transient (self-injury present at *either* T_1_ or T_2_; *N* = 13, mean (*SD*) age in years = 9.54 (*3.93*), % male = 84.6) and persistent (self-injury present at both T_1_ and T_2_; *N* = 21, mean (*SD*) age in years = 10.81 (*4.26*), % male = 81.0). The small sample size in the transient group prevented an analysis of behavioural and demographic variables associated with the onset or remission of self-injury. In order to identify putative risk markers, comparisons were made between T_1_ data for these three groups on any variables for which differences (at *p* < 0.05) had been obtained between the self-injury and non-self-injury samples at *either* T_1_ or T_2_.

**Fig. 1:**
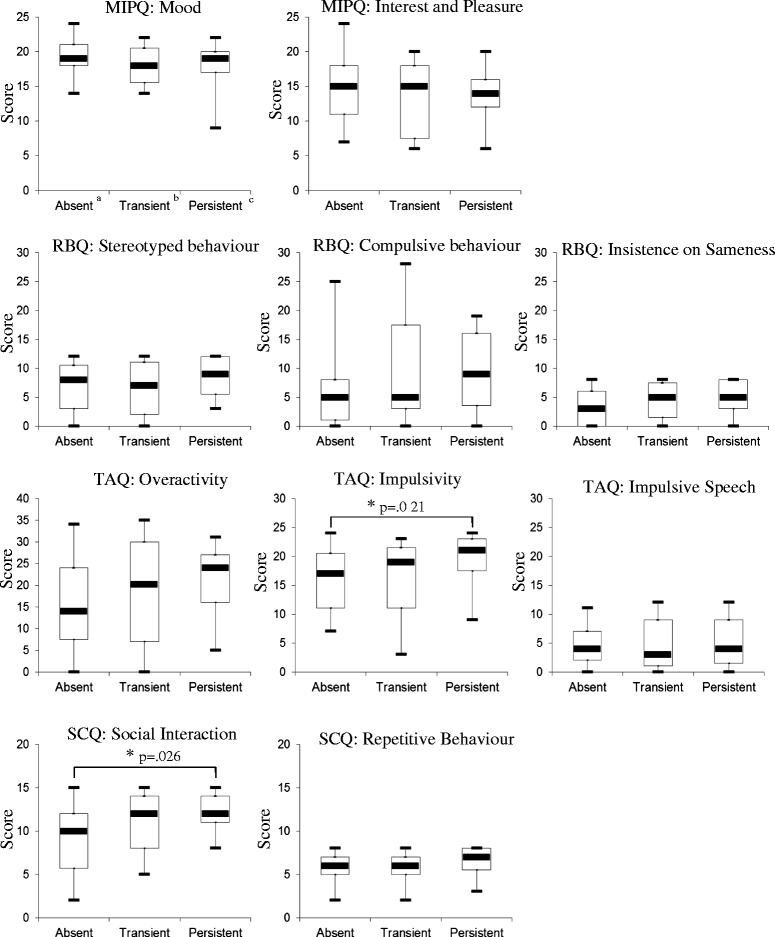
MIPQ, TAQ, RBQ and SCQ subscale scores for absent, transient and persistent self-injury groups. *a* self-injury absent at *both* T_1_ and T_2_, *b* self-injury present at *either* T_1_ or T_2_, *c* self-injury present at *both* T_1_ and T_2_

Fisher’s exact tests revealed that there were no significant differences between the three groups at T_1_ for speech (*p* = 0.059) or levels of ability (*p* = 0.171). Figure 1 displays the median, maximum and minimum scores and significant differences between groups on measures of behavioural characteristics.

The results reveal that across the majority of subscales for behavioural correlates, there was a broad trend towards the absent group having the lowest scores and the persistent group obtaining the highest scores, with the transient group falling between these two points. Significant differences between the groups were identified for Impulsivity (Kruskal-Wallis *χ*^2^ (2) = 7.33, *p* = 0.021 and Social Interaction (Kruskal-Wallis *χ*^2^ (2) = 7.49, *p* =0.026). In both cases, pairwise post hoc tests revealed significant differences between scores in the absent and persistent self-injury groups.

## Discussion

The persistence of the presence, topography and severity of self-injury in individuals with ASD was evaluated in this study. Additionally, behavioural and demographic variables associated with persistent self-injury in ASD were delineated. The recruitment of a demographically representative sample at T_2_, and the utilisation of standardised measures, strengthens the validity of the study. The use of an ASD screen at T_1_ increases the external validity of the study and ensures robust results were obtained from the sample. This study provided the first longitudinal assessment of behavioural differences between clearly differentiated groups with absent, transient and persistent self-injury and therefore the findings have significant clinical utility. The results of the study revealed self-injury to be persistent in presence, topography and severity in individuals with ASD. The demographic and behavioural variables associated with the presence of self-injury at T_1_ were revealed to be associated with self-injury at T_2_. Individuals with persistent self-injury evidenced significantly higher levels of impulsivity and impairments in social interaction at T_1_ compared to those with absent self-injury.

The results of this study revealed that self-injury was persistent over 3 years in 77.8 % of those who showed self-injury at T_1_. This finding supports data collected in populations with intellectual disability, where the persistence of self-injury has been reported at between 71 and 84 % [22, 23]. The results in this study differ from those reported in populations with ASD where self-injury was found to decrease significantly over time [5, 28]. This difference is likely due to the fact that samples assessed in previous research studies were recruited from clinical services and therefore more likely to receive interventions to reduce self-injury and/or were young samples where self-injury had not yet become established. The results from this study indicate that self-injury in ASD is persistent and stable over time, suggesting that intervention with younger children, where self-injury has a shorter reinforcement history and is less physically damaging during intervention, may be beneficial as the behaviour is unlikely to stop or decrease with time. The results extend previous research in populations with ASD by demonstrating that the topographies of self-injury in this sample were also persistent across time. This may suggest that once established, specific forms or topographies of self-injury do not change substantially over time. However, the difference in ‘hitting self with body’ did approach significance and this topography was also the most persistent form of self-injury. Further research in a larger sample is warranted to investigate whether this topography of behaviour may provide an indication that self-injury is likely to persist. Interestingly, the severity of self-injury was found to be stable across time, indicating that although self-injury did not improve, it also did not increase in parameters of severity. However, these results must be interpreted with caution. Although this sample was not drawn from a clinical population, the sample was recruited from a parent support group. It is plausible that the families included may have been receiving greater levels of behavioural support and advice than families not enrolled in a support group.

The results also revealed that the majority of variables that were associated with the presence of self-injury at T_1_ were also associated with self-injury at T_2_. Being non-verbal and having lower mood and higher levels of stereotyped behaviour, compulsive behaviour, insistence on samenesss, overactivity, impulsivity, impairments in social interaction and repetitive behaviour were all associated with the presence of self-injury. There were no significant changes in the total sample over time in any of these variables. The stability of these variables and their consistent association with self-injury is important for both pragmatic and theoretical reasons. Pragmatically, the results support the utility of delineating variables that separate those individuals with ASD for whom self-injury is likely to be persistent from those with absent self-injury. The preliminary results in this study indicate that there are stable variables associated with self-injury over time which might be considered putative risk markers for persistent self-injury.

The identification of these stable variables also informs theoretical models of self-injury in ASD. The consistent association between self-injury and ability (as evidenced through adaptive skills and speech) supports findings from applied behaviour analysis, where self-injury is identified as an operant functional behaviour [49]. It is plausible that self-injury is more likely to become socially maintained in individuals with lower levels of ability, where alternative communicative strategies are limited. Secondly, the results of this study demonstrate that higher levels of ASD phenomenology are consistently associated with the presence of self-injury, replicating findings from other studies [2]. This extends the idea, identified in previous research of ASD diagnosis as a risk marker for self-injury [36], to a more refined understanding that a greater presence or severity of ASD phenomenology is associated with the behaviour. As ASD severity is known to associate with severity of intellectual disability, these results may also highlight a role for intellectual functioning as a putative correlate of self-injury in ASD. Finally, the consistent association between low mood and self-injury replicates findings from previous studies [50]. It is possible that low mood and self-injury are indicative of depression; however, there is emerging evidence that low mood and self-injury may both be underpinned by untreated pain [19]. This requires further exploration.

The results of this study provide further evidence of compromised behavioural inhibition (as evidenced through compulsive behaviour, overactivity and impulsivity) as a behavioural correlate of self-injury. Impulsive and compulsive behaviours, such as those described in this study, and seen in individuals with attention deficit hyperactivity disorder (ADHD) have been hypothesised to be underpinned by delayed development of behavioural inhibition [51]. This impaired behavioural inhibition is suggested to reduce an individuals’ ability to stop a pre-potent response to a stimulus and to terminate an ongoing response. The growing evidence associating overactivity, impulsivity, compulsive behaviours and self-injury in ASD lends support to a theory of deficits in behavioural inhibition contributing to the development and maintenance of self-injury [3]. The presence of preserved or compromised behavioural inhibition may provide a complementary explanation to that offered in the operant literature, as to why self-injurious behaviours in young typically developing children drop out of their behavioural repertoire, whereas these behaviours persist in children with intellectual disability and ASD [52]. This hypothesis requires further research as an explanatory model of self-injury and a potential avenue for interventions in self-injury. If causal associations were identified between deficits in behavioural inhibition and the development of persistent self-injury, then early intervention for self-injury could include proactive development of strategies to increase behavioural control in ‘at risk’ children.

The results of this study also revealed two key differences in behavioural characteristics at T_1_ between the absent, transient and persistent self-injury groups at T_2_. Individuals with persistent self-injury evidenced significantly higher levels of impulsivity and difficulties in social interaction at T_1_. These behavioural characteristics represent important risk markers for persistent self-injury, evident over a 3-year period, and highlight behaviours which could be evaluated further in young children to identify whether these same variables predict the emergence of self-injury. The study has demonstrated that self-injury is worryingly stable in both presence and severity but that it is possible to identify variables which are consistently associated with the presence of self-injury and two key variables, of impulsivity and deficits in social interaction which are associated with persistent self-injury over time. Further research is now required, in younger and larger samples, to identify whether these behavioural risk markers predict the onset and severity of self-injurious behaviour. If they do, then the plausibility of identifying those with ASD most at risk of developing self-injury, and consequently the evidence base from which to develop an early intervention strategy, are strengthened. These data could be used to stratify the level of ‘risk’ an individual carries for the development of persistent self-injury, and thus proactive intervention using existing efficacious behavioural interventions could be targeted to those ‘high risk’ individuals at an earlier stage in the development of self-injury.

The study is limited by the relatively small sample size recruited at T_2_. The small sample prevented investigation of variables associated with, and predictive of, incidence and remission of self-injury. However, the validity of the results is strengthened by the utilisation of an ASD screening measure at T_1_ to ensure a homogenous sample. An additional limitation of the study is the under-representation of individuals with self-injury at the T_2_ data collection. It is possible that this under-representation may have biased the persistence and remission rates, and/or the associations between self-injury and the behavioural correlates, and thus may limit the external validity of the findings. However, no other behavioural or demographic variables differed between the two samples and at both time points the identified prevalence of self-injurious behaviour was in line with other robust estimates in the literature, suggesting that the sample is still representative of the wider ASD population. Similarly to previous longitudinal studies investigating persistence, this study did not collect data on pharmaceutical and behavioural treatment for self-injury in the sample over time [5, 23]. Thus, it was not possible to evaluate the extent to which the identified persistence rate is the natural course of self-injury or a result of limited access or efficacy of interventions. Likewise, the identified remission rates and consequent prevalence of individuals with ASD who *do not* show self-injury may be due to the implementation of effective treatments. It would have been beneficial to collect these data to allow further understanding of the factors which effect persistence of self-injury. Future studies should seek to include assessment of intervention treatments, alongside the delineation of behavioural and demographic correlates, in order to build a more complete model of the trajectory of self-injury in individuals with ASD. These data should be collected in older children, adolescents and adults with ASD to detail the progression of self-injury in older cohorts.

In summary, the results have revealed that self-injury is a persistent and stable behaviour over 3-years in individuals with ASD and that risk markers of impulsivity and deficits in social interaction are associated with persistent self-injury over time.

## Conclusions

The results of the present study demonstrate that self-injurious behaviour in ASD is highly persistent over 3 years. The presence of impulsivity and deficits in social interaction are longitudinal risk markers for persistent self-injury in this population. These results lend support to models implicating behaviour dysregulation and ‘ASD type’ deficits in the development and maintenance of self-injury. The identification of these risk markers can be usefully applied to target early intervention approaches towards those individuals who are most likely to show persistent self-injury.

## Abbreviations

ASD: autism spectrum disorder
CBQ: Challenging Behaviour Questionnaire
MIPQ: Mood, Interest and Pleasure Questionnaire
RBQ: Repetitive Behaviour Questionnaire
SCQ: Social Communication Questionnaire
TAQ: The Activity Questionnaire
